# The Efficacy of Amifostine against Multiple-Dose Doxorubicin-Induced Toxicity in Rats

**DOI:** 10.3390/ijms19082370

**Published:** 2018-08-12

**Authors:** Vesna Jaćević, Viktorija Dragojević-Simić, Željka Tatomirović, Silva Dobrić, Dubravko Bokonjić, Aleksandra Kovačević, Eugenie Nepovimova, Martin Vališ, Kamil Kuča

**Affiliations:** 1 Department of Experimental Toxicology and Pharmacology, National Poison Control Centre, Military Medical Academy, 11 Crnotravska St, 11000 Belgrade, Serbia; v_jacevic@yahoo.com; 2 Medical Faculty of the Military Medical Academy, University of Defense in Belgrade, 1 Pavla Jurišića-Šturma St, 11000 Belgrade, Serbia; vdragsim@gmail.com (V.D.-S.); zeljka.tatomirovic@gmail.com (Ž.T.); silva.dobric@gmail.com (S.D.); bokonjic.dubravko@gmail.com (D.B.); alexandra.kova@gmail.com (A.K.); 3 Department of Chemistry, Faculty of Science, University of Hradec Kralove, Rokitanského 62, 50003 Hradec Králové, Czech Republic; evzenie.n@seznam.cz; 4 Centre for Clinical Pharmacology, Military Medical Academy, 11 Crnotravska St, 11000 Belgrade, Serbia; 5 Institute for Pathology, Military Medical Academy, 11 Crnotravska St, 11000 Belgrade, Serbia; 6 Institute for Scientific Information, University of Defense in Belgrade, 1 Pavla Jurišića-Šturma St, 11000 Belgrade, Serbia; 7 Department of Neurology, Charles University in Prague, Faculty of Medicine in Hradec Kralove and University Hospital, Simkova 870, 50003 Hradec Králové, Czech Republic; Valismar@seznam.cz

**Keywords:** doxorubicin, amifostine, bone marrow, hepatotoxicity, nephrotoxicity, rats

## Abstract

Amifostine is well known cytoprotector which is efficient when administered before a wide range of antineoplastic agents. The aim of our study was to investigate amifostine effects on doxorubicin-induced toxic changes in rats. Amifostine (75 mg/kg ip) was given 30 min before each dose of doxorubicin (cumulatively 20 mg/kg ip, for 28 days). The animals’ whole-body, liver, and kidney weight, serum biochemical examination, as well as microscopic examination of bone marrow, peripheral blood, liver, and kidney, were done on day 56 of the study. Hepatic and renal alterations were carefully quantified by semiquantitative grading scales—hepatic and renal damage score, respectively. In amifostine-pretreated rats, the number of peripheral blood leukocytes was significantly higher in comparison to doxorubicin-only treated group, preferentially protecting neutrophils. In the same group of rats, hepatic and renal alterations associated with polymorphonuclear cell infiltrates were significantly less severe than those observed in animals receiving only doxorubicin. Our results showed that amifostine successfully protected rats against multiple-dose doxorubicin-induced toxicity by complex, and still not fully elucidated mechanisms of action.

## 1. Introduction

Doxorubicin (DOX) is an anthracycline antibiotic that is used as an antineoplastic agent in hematological, as well as in solid malignancies, due to its high antitumor efficacy [1,2,3,4,5,6,7]. However, its clinical use is limited by the myelosuppressive effects and development of irreversible cardiotoxicity, as well as its ability to cause cancer cell resistance during therapy [8,9]. Stomatitis, gastrointestinal (GI) disturbances, and alopecia are common, but reversible.

It is well known that DOX is able to interfere with a number of biochemical functions within cells, but the precise molecular pathogenesis of both therapeutic and toxic effects are still controversial [4,5]. There are numerous experimental models concerning these subjects, which showed DOX-induced bone marrow toxicity, cardiotoxicity, hepatotoxicity, nephrotoxicity, and toxic effects on the GI tract (GIT), reproductive system, and nervous system [4,9,10,11,12,13,14,15]. DOX acts as a strong inhibitor of DNA duplication and transcription, with the most obvious consequences on tissues with strong proliferative potential, such as bone marrow, GIT, and the reproductive system. On the other hand, DOX, by virtue of its quinone group, generates free radicals in vitro and in vivo, which is significantly stimulated by its interaction with iron. Moreover, oxidative stress, apoptosis, and inflammation can be taken as possible mechanisms of DOX multiple-organ toxicity, such as cardiotoxicity on the first place [5,12,15,16,17,18,19].

As far as the liver is concerned, DOX is metabolized predominantly by liver microsomal enzymes and cytoplasmic reductase [20,21] to the major metabolite doxorubicinol, and several hepatotoxic aglycone metabolites [22,23]. Drug hepatotoxicity may ensue through free-radical formation and generation of reactive oxygen species (ROS), such as superoxide anions (O_2_^−^), hydroxyl radicals (OH·), and hydrogen peroxide, which induce lipid peroxidation and protein oxidation [5,24], and impair endogenous antioxidant defenses [25]. Since hepatic cell membrane lipids are also susceptible to DOX-induced oxygen radical injury, peroxidation continues autocatalytically, resulting in structural and functional alterations in the hepatic tissue. In that case, irreversible alterations lead to hepatocyte apoptosis or necrosis, and intensive increase of hepatic enzymes in the blood, primarily alanine aminotransferase (ALT) and aspartate aminotransferase (AST) [9]. It is also believed that the DOX-induced nephrotoxicity may be mediated through free radical formation, iron-dependent oxidative damage of biological macromolecules, membrane lipid peroxidation, and protein oxidation [15,19,26]. Experimental animals that received DOX developed focal segmental glomerulosclerosis, followed by massive proteinuria, which is a certain nephrotic syndrome severity marker [10,27]. Further, progressive loss of renal function was tightly associated with increased levels of creatinine, uric acid, and blood urea nitrogen [11,28].

It is considered that the successful prevention or treatment of these DOX-induced harmful effects depends on antioxidative potential of different cytoprotective agents and their ability to react with highly reactive oxygen species (ROS) [12,13,26,28,29,30,31,32].

Amifostine (AMI) is a thiophosphate derivative of cysteamine, which, if administered before cytotoxic chemotherapy, provides protection of various normal tissue, without attenuating their antitumor response [33,34,35,36]. It is actually a prodrug that cannot protect tissues until dephosphorylated by alkaline phosphatase in the plasma membrane to the active metabolite, WR-1065. Once inside the cell, its protective effects appear to be mediated by scavenging free radicals, hydrogen donation, induction of cellular hypoxia, liberation of endogenous nonprotein sulfhydryls (mainly glutathione) from their bond with cell proteins, the formation of mixed disulfides to protect normal cells, etc. It is a broad spectrum cytoprotector: it protects bone marrow against harmful effects of ionizing radiation, as well as cyclophosphamide, nitrogen mustard, melphalan, mitomycin C, 5-fluorouracil, carboplatin, and cisplatin [34,35,36,37,38,39]. Protection from cisplatin nephrotoxicity and ototoxicity has been shown, as well as protection of peripheral neural tissue from cisplatin, paclitaxel, and vincristine toxicity. Cardioprotective effects of AMI in rats treated by a large single dose of DOX was evaluated [12], as well as with low unitary doses of DOX which, when cumulatively administered, eventually lead to progressive cardiomyopathy [33].

Although DOX toxic effects on the liver and kidney are probably mediated initially by highly reactive free radical formation, we hypothesized that final irreversible changes are the result of inflammatory reactions involving cells of bone marrow origin, like was previously demonstrated in the rat model of chronic cardiac toxicity induced by DOX [33].

Therefore, our aim was to investigate AMI effects on DOX-induced toxic changes in bone marrow, liver and kidney, and their contribution to the significantly better survival of the rats treated by multiple, low doses of DOX.

## 2. Results

### 2.1. General Health Condition, Body Weight, Liver and Kidney Weight of the Experimental Animals

Visible signs of general weakness, with hemorrhagic diarrhea, adynamia, and hair loos, have been seen in only the DOX-treated group. Also, these animals were extremely anxious to all kinds of stimuli, especially 2 days after the application of DOX.

**Figure 1:**
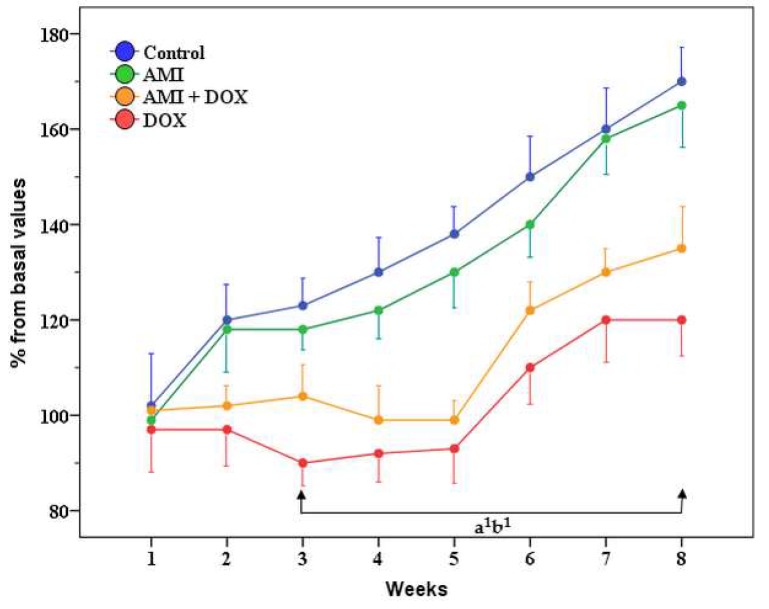
The influence of amifostine (AMI) and/or doxorubicin (DOX) on the body weight of rats during the 8 weeks of the study. The results are expressed as a percentage from the control group. **a^1^**—*p* < 0.05 for DOX compared with the control group; **b^1^**—*p* < 0.05 for AMI + DOX compared with the control group.

Rats treated with AMI-only and control group showed a steady weight gain, especially after the first week of treatment (Figure 1). Animals treated with DOX-only lost body weight during the first 3 weeks of treatment, and then showed slow weight gain, but it was significantly lower (*p* < 0.05) in comparison to control throughout the remaining 5 weeks of the study. The body weight of rats protected with AMI was also lower compared with the control group, especially during the last 5 weeks of experiment, but it was above that of the DOX-only treated group, although not significantly, especially during the last 3 weeks of the study.

Four weeks after the last treatment, there were no statistical differences between the relative liver and kidney weights in rats pretreated with AMI, in comparison to control animals (Table 1). However, the relative liver and kidney weight of rats that received only DOX were significantly higher in comparison to the control group. Multiple applications of AMI only did not significantly affect these parameters in experimental animals.

### 2.2. The Effects of Different Treatment on Biochemical Parameters in the Blood Serum of the Experimental Animals

**Figure 2:**
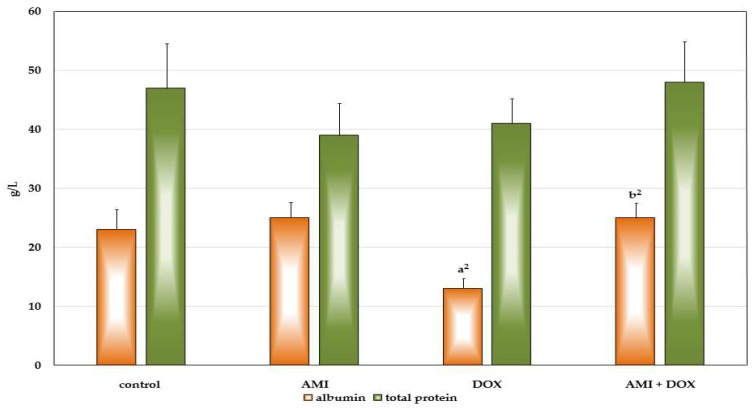
The influence of the treatment with amifostine and/or doxorubicin on albumin and total protein serum content 28 days after receiving last treatment. **a^2^**—*p* < 0.01 in comparison to the control group; **b^2^**—*p* < 0.01 in comparison to the DOX-only treated group.

Multiple applications of DOX decreased the total protein serum content in the rats 4 weeks after the last application, primarily as a result of a highly significant decrease of albumin (Figure 2). On the other hand, in the rats pretreated with protector AMI, total serum protein content was higher than in the DOX-only treated group, due to a highly significant increase of albumin, whose content was not different in comparison to control animals. AMI itself did not affect neither total protein content nor albumin content of the serum of the experimental animals.

**Figure 3:**
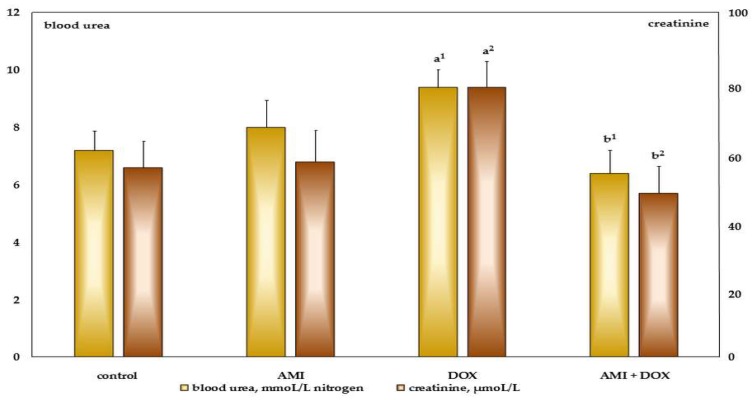
The influence of the treatment with amifostine and/or doxorubicin on blood urea nitrogen and creatinine serum content 28 days after receiving last treatment. **a^1^**, **a^2^**—*p* < 0.05, 0.01 in comparison to the control group; **b^1^**, **b^2^**—*p* < 0.05, 0.01 in comparison to the DOX-only treated group.

The significant increase of serum level of blood urea nitrogen and creatinine was observed in the group of rats treated with DOX (Figure 3). However, previous treatment of AMI in experimental animals completely prevented these changes in the rats treated with antineoplastic agent DOX. The fact—also very important for this investigation—is that the multiple application of AMI itself does not affect serum blood urea nitrogen and creatinine levels in the experimental animals.

### 2.3. The Effects of Different Treatments on Granulopoiesis and Erythropoiesis in the Bone Marrow and Peripheral Blood of Experimental Animals

The percentage of myeloblasts, promyelocytes, and myelocytes in the group of DOX-only treated rats was not significantly different from the control animals (Table 2). However, the percentage of metamyelocytes and non-segmented granulocytes was significantly decreased in this group of rats, in comparison to the control one, but pretreatment with AMI effectively protected these subpopulations of immature forms of leucocytes. In the group of rats pretreated with AMI, the total number of neutrophil granulocytes was also significantly increased in comparison to DOX-only treated group (Table 2), in accordance with the findings of differential blood count in peripheral blood 28 days after receiving the last treatment (Table 3).

The relative number of lymphocytes has not been significantly changed in the bone marrow, although it has tendency to be decreased in all groups of treated rats in comparison to control, especially in the peripheral blood of rats pretreated with AMI (Table 3).

Also, pretreatment with AMI significantly decreased the percentage of eosinophil leucocytes in bone marrow, which was increased due to the effect of DOX (Table 2). The relative number of mast cells were significantly lower in both DOX-only treated rats, as well as in AMI-pretreated animals in comparison to control group.

**Figure 4:**
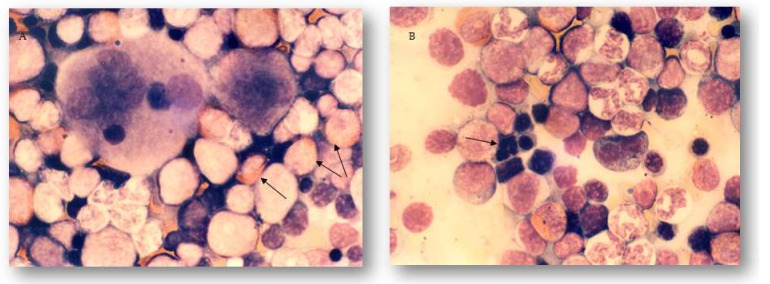
Light micrographs of the bone marrow of rats 28 days after receiving last treatment. May-Grünwald–Giemsa stain, magnification 1000×. (**A**) Eosinophilia in the bone marrow of the DOX-only treated rats 28 days after receiving last treatment marked with arrows. (**B**) Sign of dyserythropoiesis in the bone marrow of the DOX-only treated rats 28 days after receiving last treatment marked with arrow.

A careful analysis of the bone marrow in DOX-only treated rats showed marked eosinophilia and dyserythropoiesis, which was not present in AMI-pretreated animals (Figure 4A,B).

Multiple applications of AMI-only affected neither of the examined hematological parameters, both in the bone marrow and peripheral blood, of the experimental animals 28 days after the last application of this protector.

### 2.4. Pathohistological Examination of Experimental Animal Tissue Alterations

#### 2.4.1. Hepatic Alterations

**Figure 5:**
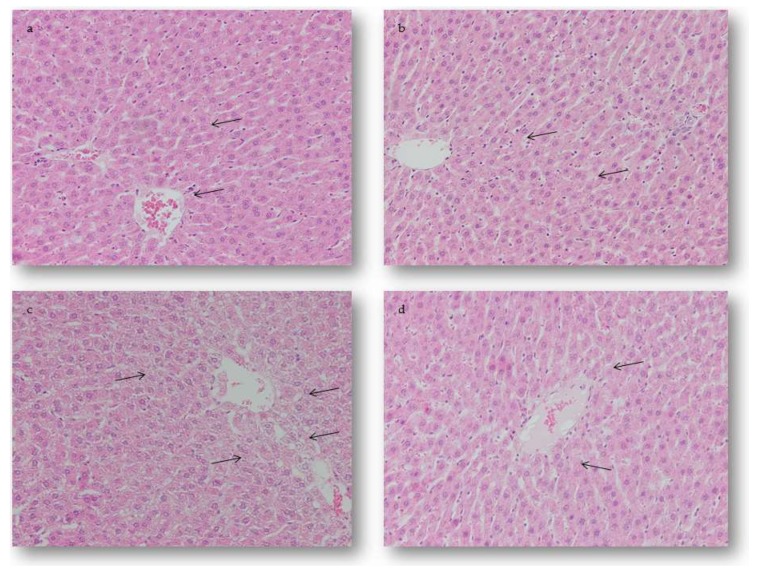
Light micrographs of the hepatic lesions of rats 28 days after receiving last treatment. Hematoxylin and eosin (H&E) stain, magnification 200×. (**a**) Normal histological structure of the hepatic tissue marked with arrow. (**b**) The AMI-treated group, mild oedema in the sinusoidal spaces marked with arrow. (**c**) The DOX-treated group, severe intracellular degeneration marked with arrow. (**d**) The AMI + DOX-treated group, mild intracellular degeneration marked with arrow.

Microscopic examination of the hepatic tissue sections of the control animals has shown normal histological architecture without changes during the whole study period (Figure 5A). On the hepatic sections of the rats treated with AMI only, mild oedemas in the sinusoidal space were seen on day 28 after receiving last treatment (Figure 5B). On the other hand, in hepatic sections of the rats treated with DOX only, numerous intracytoplasmic vacuoles were seen, while the usual radial arrangements of the liver parenchyma were segmentally lost. Moderate oedema and hyperemia were expressed in all sinusoidal spaces. Focal hemorrhages were localized primarily perivascularly, and partly in the periportal space of the hepatic lobule. Hypertrophic Kupffer’s cells could be seen in the sinusoidal spaces. Almost all hepatocytes were oedematous with marked intracellular degeneration. Also, the majority of the blood vessels were dilated, with discontinued basal membranes seen on some sections, surrounded by an accumulation of polymorphonuclear cells (PMNCs) (Figure 5C). The application of AMI prior to DOX significantly attenuated the degenerative and vascular changes caused by this chemotherapeutic agent. In this group, the appearance of small, individual vacuoles was observed in a limited number of hepatocytes in the central part of the lobules, while the radial arrangement of the hepatocytes was largely sustained with the presence of mild oedema and hyperemia. Also, the sinusoidal spaces were slightly extended, with increased number of individual Kupffer’s cells. Additionally, all blood vessels were just slightly dilatated without alterations in their basal membranes, and were surrounded by individual PMNCs (Figure 5D).

#### 2.4.2. Renal Alterations

**Figure 6:**
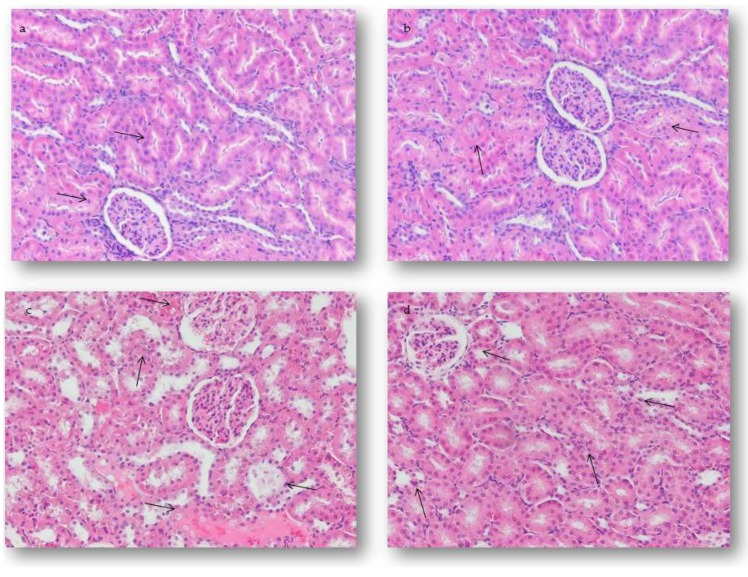
Light micrographs of the renal lesions of rats 28 days after receiving last treatment. H&E stain, magnification 200×. (**a**) Normal histological structure of the renal tissue marked with arrow. (**b**) The AMI-treated group, mild oedema in the renal epithelial cells marked with arrow. (**c**) The DOX-treated group, extensive vascular and degenerative changes in the renal tissue associated with atrophy of some glomeruli marked with arrow. (**d**) The AMI + DOX-treated group, normal glomeruli, and mild PMNCs infiltration of the renal cortex and medulla marked with arrow.

Microscopic examination of the renal tissue sections of the control animals has shown normal histological architecture without changes (Figure 6A). On the renal micrographs of the rats treated with AMI only, mild oedema in the renal epithelial cells was seen on day 28 after receiving the last treatment (Figure 6B). Four weeks after receiving the last treatment in rats treated with DOX only, moderate vacuolar changes in renal tubular epithelial cells, extensive vascular changes, and atrophy of some glomeruli were seen. In some tissue sections, these lesions progressed to degeneration and reduction of tubular epithelial cells. However, in parallel with renal tubular epithelium disappearance, there was a corresponding increase in representation of interstitial connective tissue. Also, the initial signs of perivascular fibrosis were seen in some tissue samples. Marked thickening of the juxtaglomerular arteries was associated with loss of some glomeruli at the end of the study. Furthermore, in DOX-only treated rats, the majority of the blood vessels were dilated, with discontinuous basal membranes seen on some sections, surrounded by an accumulation of PMNCs both in the renal cortex and the medulla (Figure 6C). Pretreatment with AMI decreased the frequency and severity of described renal alterations in experimental animals (Figure 6D).

### 2.5. Semiquanitative Analysis of the Experimental Animal Tissue Alterations

#### 2.5.1. Quantification of Hepatic Alterations

Semiquantitative pathohistological analysis confirmed that the AMI given to rats as a pretreatment to DOX diminished degenerative and vascular changes in hepatic tissue caused by this antineoplastic agent, 4 weeks after the last treatment (Table 4). In this group of animals, the hepatic damage score (HDS) was significantly lower than those established in the group treated with DOX only (3.62 ± 0.51). AMI, applied alone, in a single dose of 75 mg/kg ip, caused mild structural alterations defined as HDS of 1.37 ± 0.75, while HDS of control animals was 0.25 ± 0.46. The most intensive degenerative and vascular alterations, scored as 4.62 ± 0.51, were seen in the group treated with DOX on day 28 after receiving the last treatment.

#### 2.5.2. Quantification of Renal Alterations

Four weeks after the last treatment, pretreatment with AMI significantly decreased the intensity of renal alterations caused by DOX (Table 5). In this experimental group, pretreatment resulted in significant decrease of renal damage score (RDS) (3.25 ± 0.46) compared with the group of animals treated with DOX only (4.75 ± 0.46). Multiple applications of AMI, in a single dose of 75 mg/kg ip, led to minimal renal alterations (1.50 ± 0.53), while in the control group of animals, RDS was 0.25 ± 0.46 on day 28 after receiving the last treatment.

## 3. Discussion

Our results indicate that AMI, administered in a dose of 75 mg/kg, before each dose of DOX, significantly attenuated the effects of this antineoplastic agent (1.25 mg/kg ip, four consecutive days during the week, for four weeks, cumulatively, 20 mg/kg body weight, for 28 days) in rats. Moreover, the rats were monitored four weeks after the last administration of DOX, since our previous examination showed that the obtained survival of experimental animals (AMI significantly increased the percentage of survived rats in comparison to the group received DOX-only, 90.4% vs 63.6%, respectively) enabled further, more detailed research [33].

We observed, as well as Rossi et al. [40], significant reduction of rats’ body weight, starting from the third week after application of DOX and, although the body weight of AMI-treated rats was also significantly lower in comparison to the control group, it was still higher than in the group treated with DOX only, especially in the last three weeks of the study.

When DOX was administered in small doses, over a period of weeks, depending on its cumulative dose, a significant decrease in the body weight compared to the control group was observed [13,41]. This was due to the loss of appetite and massive lesions in the epithelium of the GIT that prevented food intake and its utilization [13,42]. However, DOX-induced cardiomyopathy and nephropathy found in mice, rats, and dogs, after repeated administration, was the consequence of its toxic activity, and the main cause of death of these animals [13,26,33,41,42,43,44]. Namely, in the peritoneal and pleural cavities of rats, massive exudates were observed, which was not the case in animals protected with AMI. The ventral subcutaneous oedema, hydropericardium, hydrothorax, or ascites were consequences of congestive heart failure that develops in these animals [13,33]. Moreover, in animals treated with DOX, the relative liver and kidney weight was significantly higher than in the control group, which is in accordance with our results. These changes were due to congestive DOX cardiomyopathy. On the other hand, the relative liver and kidney weight in AMI-protected rats were similar to the control values. This finding supports the statement that the protective effects of AMI are largely based on its protection against cardiotoxic action of DOX [13,33].

In addition, in the experiments in which AMI was used as a protector against toxic effects of DOX in mice, this protector reduced mortality and protected mice from the hematotoxicity of DOX [45]. Therefore, we also investigated the efficacy of AMI as a protector against myelotoxic effects of DOX, in order to evaluate its contribution to the better survival and wellbeing of the animals treated with multiple doses of this antineoplastic agent.

In the bone marrow, good cellularity was established in all group of experimental animals, as well as the presence of individual hematopoiesis cell lines. Since the last administration of DOX was performed four weeks prior to the end of the study, there was sufficient time for bone marrow of animals treated with DOX to recover [14]. However, detailed morphological analysis of the bone marrow, as well as established percentage of all granulopoetic cell lines (precursors and mature forms), confirmed the statement that DOX acts as a strong inhibitor of DNA duplication and transcription [46]. This is manifested within the erythrocyte cell line as dyserythropoiesis, while in the granulocytic cell line, the presence of a delayed type of maturation of the neutrophil granulocytes was observed, what was substantiated by the statistically significant difference in comparison to control animals. These changes were found in the DOX-only treated group, but not in the AMI-protected animals, and these findings support the effective protection of these two cell lines of hematopoiesis. The obtained results of the analysis of peripheral blood are the reflection of what is ongoing in the bone marrow of all experimental groups. It was also noted that AMI significantly reduced the number of eosinophils in the bone marrow compared with animals treated with DOX only. This eosinophilia most likely reflected the allergic reactivity of the rat bone marrow to DOX.

The pronounced monocytosis was established in the peripheral blood of both DOX only and AMI + DOX treated groups. This monocytosis could be interpreted as a possible recruitment of a monocyte–macrophage system for the elimination of DOX-affected tissues. For example, data on increased migration of macrophages into the thymus are known, in conditions such as increased destruction of thymocytes, due to the action of cyclophosphamide antineoplastic agent [47]. Only the lymphocytic cell line was not protected by AMI, which was obvious from peripheral blood smears.

Previous studies showed that pretreatment with AMI not only protected animals from the hematotoxicity of different antineoplastic agents [38,45], but also in vitro, in normal human multipotent and target stem cells (multipotential progenitor cells for myeloid cells, i.e., CFU-GEMM; granulocyte-macrophage colony-stimulating factor—GM-CSF; burst-forming unit-erythroid—BFU-E) were preserved from the cytotoxic effects of doxorubicin, daunorubicin, mitoxantrone, paclitaxel, and cyclophosphamide derivatives [39,48,49]. Moreover, in in vitro conditions, thiols, especially AMI, stimulated the growth of human stem cells of the bone marrow by inhibiting apoptosis caused by the lack of appropriate hematopoietic growth factor (CSF) [48,50,51]. In the same experimental conditions, interleukin 1 (IL-1) was able to induce multiple increases in colony stimulating factor (CSF) production, either a single hematopoietic line or multipotent stem cells [52,53]. Also, administration of IL-1 to mice caused the appearance of an increased number of large bone marrow cell subpopulation that entered into S, G2, and M phase of cell cycle, and increased the number of cultured cells that were reactive to GM-CSF [54]. The other thiol, diethyldithiocarbamate, protected the bone marrow of mice from antineoplastic agent toxicity by increasing the secretion of a series of cytokines, such as IL-1β and hematopoietic growth factors for granulocytes [55]. It has also been shown that the administration of IL-1 alone prevents significant depletion of myeloid and erythroid bone marrow cells caused by high doses of DOX [56]. However, during in vitro conditions, normal human multipotent and target stem cells in the presence of AMI and WR-1065 proliferated three times as much as in the presence of IL-1 and IL-3, which also acted on hematopoietic stem cells [48]. Thus, the protective effect of AMI in our experiment is probably based on the “cytokine-like” stimulation of the stem cells of the bone marrow, maybe by inhibiting its apoptosis, or by DNA protection and repair acceleration [34,35,36,50,51,52].

Serum biochemical examinations revealed that multiple application of DOX led to decrease in the serum total protein content, primarily albumin, and significant increases in blood urea nitrogen and creatinine content, while administration of AMI before DOX completely prevented these changes. The same biochemical profile of the results were obtained in DOX-induced nephropathy, as a model of chronic progressive glomerular disease [57,58]. Significant hypoalbuminemia, and increased levels of blood urea nitrogen and creatinine in the blood of these rats were associated with an increase in serum triglyceride and cholesterol levels, as well as reduced levels of iron and massive proteinuria. Moreover, reduced protein intake and decreased utilization, partly as a result of mentioned loss of appetite and massive lesions in the epithelium of the GIT, contribute to further development of hypoalbuminemia, edema, weight loss, and generally poor condition of animals [13,42]. Furthermore, insufficient synthesis of serum proteins, including albumin, due to toxic effects on the liver, could also contribute to this bad medical condition of the DOX-treated animals. However, pretreatment with AMI significantly attenuated DOX-induced cumulative effects and maintained protein, blood urea nitrogen, and creatinine serum content at a basal level. Multiple application of AMI only did not cause any changes in the protein, blood urea nitrogen, and creatinine levels in the serum.

Moreover, pretreatment with AMI significantly reduced HDS and RDS in comparison to the rats given DOX only. It was shown that the earliest and most frequent changes in the rat liver and kidney after application of DOX, in cumulative dose, were the consequences of its toxic effect on the hepatocyte, endothelial cells, and renal tubular epithelial cells [18,32,59]. The current understanding of molecular mechanisms underlying DOX-induced hepatic and renal cell type of death, both apoptosis and necrosis, implies excessive toxic and ischemic injuries of the hepatocytes, renal epithelial cells and vascular endothelium in the experimental animals [18,32,59,60], which was substantiated by our results. Chemotherapeutic agents may cause direct hepatic toxicity, and alteration of its function may affect drug metabolism and cause an increased risk of general toxicity [61]. Namely, since the major route of DOX elimination is the hepatobiliary system, i.e., DOX is metabolized predominantly by the liver microsomal enzymes and cytoplasmic reductase, and excreted mostly in bile, toxicity to recommended doses of DOX is enhanced by impairment of liver function, since it results in slower elimination, and consequently, increased retention and accumulation in plasma and tissues [20,21,61]. Therefore, increased concentration of DOX not only has recurrent direct hepatotoxic effects, but also potentiated other toxic effects, including cardiotoxicity, which mostly limits its clinical use, as well as nephrotoxicity, which is species-specific in rats. Generally, in heart failure, there is a compensatory increase in blood volume that serves to increase stroke volume, but also, due to sympathetic activation, reduced renal perfusion and decreased urine output with the retention of water [62,63]. This, in combination with sympathetic activation of the kidney, stimulates the release of renin, thereby activating the renin–angiotensin–aldosterone system. There is also an increase in circulating vasopressin, and the final outcome of humoral activation is an increase in renal retention of sodium and water. It raises venous pressures and accumulation of venous blood, which can lead to generalized oedema. The accumulation of venous blood, rich in CO_2_, provokes hypoxic damage of the endothelial cells in capillary walls, which also contributes to further emerging of oedema [64,65]. An already weakened heart is unable to increase cardiac output, so further increase in the intravascular volume and venous pressure leads to additional fluid release into the tissues. On the other hand, hepatic oedema contributes to the increased permeability of the capillary wall that has already arisen due to the damage under the influence of DOX [9,15,32,59]. Such intensive disorders of water metabolism lead to the formation of small or large vacuoles in the cytoplasm of hepatocytes, as result of extreme enlargement of mitochondria and vesiculation of endoplasmic reticulum. Nucleus alterations, often detected in hepatocytes as early signs of necrosis, were also showed in our study as hydropic degeneration, without clearly visible nuclei in the hepatocytes of DOX-treated animals. In histological micrographs in our study, irreversible damage has also been seen as a loss of physiological radial arrangement of hepatocytes, almost all of them were oedematous with marked intracellular degeneration, with concomitant hyperemia and focal hemorrhages. In our study, almost all previously described toxic and ischemic injuries, when affecting renal epithelial cells and vascular endothelium in the rats due to direct noxious effects of DOX, as well as DOX-induced heart failure, led to moderate vacuolar changes in renal tubular epithelial cells, extensive vascular alterations, and atrophy of some glomeruli in histological micrograph. In some tissue sections, these lesions progressed to degeneration and reduction of the tubular epithelial cell size. Among other pathohistological findings in DOX-only treated rats, the majority of the blood vessels were dilated, with discontinued basal membranes seen on some sections, surrounded by an accumulation of PMNCs, both in the liver and kidney.

Although the exact mechanism of DOX-induced hepatotoxicity and nephrotoxicity remains unknown, it is believed that the toxic effects are mediated by free radical formation, iron-dependent oxidative damage of biological macromolecules, membrane lipid peroxidation, and protein oxidation [15,19,24,25,26,66,67]. Moreover, nitric oxide synthase may be responsible for the reductive activation of DOX to its free radical semiquinone form, and the subsequent oxygen radical-mediated cellular damage.

In AMI-protected animals, hepatic and renal tissue alterations were significantly less severe than those observed in animals treated with DOX only. It is well known that AMI active metabolite, WR-1065, once inside the cell, is powerful scavenger of oxygen free radicals [33,34,35,36]. Previous in vitro studies using a pure chemical system demonstrated that WR-1065 was able to scavenge OH· and O_2_¯, including DOX-derived O_2_¯ generated by nicotinamide adenine dinucleotide (NADH) respiration of heart mitochondria particles [68]. Moreover, in our study, in AMI-treated group, both in the liver and kidney of the experimental animals, most of the blood vessels were just slightly dilated without prominent alterations in their basal membranes, and were surrounded by small groups or by individual PMNCs. In our previous work, we actually showed that AMI in a range of doses, from 100 to 300 mg/kg body weight, had potent anti-inflammatory activity in a model of acute inflammation in rats [29]. This mechanism of action might additionally offer protection against DOX-induced acute [12,33], and especially chronic [69] cardiotoxicity in rats. It was probably a consequence of inhibition of PMNCs infiltration, as well as main pro-inflammatory mediator production, including free radicals [12,29,33,36,59,70,71]. Therefore, we hypothesize that protective effects of AMI against hepatic and renal alterations caused by DOX are also based on its potent antioxidative and anti-inflammatory effects. Moreover, AMI itself, given in multiple doses, did not cause any irreversible changes in the liver and kidney of experimental animals in comparison to the control rats.

## 4. Materials and Methods

### 4.1. Chemicals

DOX (Adriablastina^®^) for iv administration was purchased from Pharmacia & Upjohn (Milan, Italy). Amifostine was synthesized in the Chemical Department of Military Technical Institute, Belgrade, Serbia, by an original previously described procedure [33]. AMI was prepared for administration by dissolving the substance in sterilized and apyrogenic 0.9% NaCl solution, ex tempore.

### 4.2. Experimental Animals

Experiments were performed on male Wistar rats, 6–8 weeks old (200 to 220 g) bred at the Department for Experimental Animals, Military Medical Academy, Belgrade, Serbia. The experimental animals were housed in groups of five in plastic cages (Macrolon^®^ cage type 4, Bioscape, Castrop-Rauxel, Germany) with sawdust bedding (Versele-Laga, Deinze, Belgium) certificated as having contaminant levels below toxic concentrations. The environmental conditions were controlled and monitored by a central computer-assisted system with a temperature of 22 ± 2 °C, relative humidity of 55 ± 15%, 15–20 air changes/h, and artificial lighting of approximately 220 lux (12 h light/dark cycle). The experimental animals had free access to food, commercial pellets for rats (Veterinarski Zavod Subotica, Subotica, Serbia) and tap water from municipal mains, filtered through 1.0 µm filter (Skala Green, Belgrade, Serbia).

All the above environmental conditions, as well as all the procedures adopted for housing and handling of experimental animals, were in strict compliance with the Guidelines for Laboratory Animal Welfare of the Ethics Committee for Experiments on Animals of the Military Medical Academy, Belgrade, Serbia, which were adopted and are in complete accordance with the current Guidelines for Animal Welfare approved by the European Commission.

The study protocol was approved by the Ethics Committee for Experiments on Animals issued by Military Medical Academy, Belgrade, Serbia (approved study protocol no.: 282-12/2002, 8 December 2014).

### 4.3. Experimental Design

Wistar rats were randomly divided into four experimental groups each containing 10 individuals. The animals received the following treatments: (1) Control (0.9% NaCl), (2) AMI (AMI 75 mg/kg ip) 30 min before saline (1 mL/kg ip), (3) DOX (DOX 1.25 mg/kg ip 4 times per week during 4 weeks), and (4) DOX (DOX 1.25 mg/kg ip 4 times per week during 4 weeks) + AMI (AMI 75 mg/kg ip 30 min before DOX). The total cumulative dose of DOX administered to rats (20 mg/kg ip) was estimated as being sufficient to induce progressive cardiomyopathy [72].

### 4.4. General Health Condition and Weight Changes

Mortality and general condition of the animals were observed daily throughout the whole experiment lasting 8 weeks. Body weights were recorded 4 times per week for 4 weeks during the treatment, and once a week for a further 4 weeks, until the end of experiment. Postmortem examination, and liver and kidney excision and weighing, were also done at that time. Each experimental group consisted of 10 animals.

### 4.5. Biochemical Assays

The blood samples were taken and collected in plain tubes for serum separation. Then, blood samples were allowed to clot at room temperature (25 °C) for up to 30 min, after which they were centrifuged for 10 min at 3000 rpm at 25 °C. The separated serum was assessed for hemolysis, and then stored at −20 °C until assayed. The blood serum was analyzed by the auto-analyzer ASTRA-8 (Beckman, London, UK) with the use of reagent kits. The standard parameters measured and methods adopted were as follows: total protein, albumin, blood urea nitrogen and creatinine, by end-point determination on colorimetric reaction.

### 4.6. Bone Marrow and Peripheral Blood Sample Preparation

Twenty-eight days after the last DOX and/or AMI administration, ten animals from each experimental group were used for bone marrow and peripheral blood samples. For the preparation of the blood smear and differential blood count, blood was taken from tail vein of a rat by using syringes with needle. A small drop of the blood smears was prepared on clear glass slides, air-drying (2–4 h) at room temperature. Finally, slides were stained using routine May-Grünwald–Giemsa staining method.

Then, animals were euthanized under light ether anesthesia. Both femurs of the rat were removed through the pelvic bone, and the femur bone was set free from the extra muscles. Then, epiphyses were cut off and bone marrow plugs were flushed out with a needle and aspirated into the syringe with small amount of fetal calf serum. The cell suspensions were centrifuged for 10 min at 1000 rpm, and sedimented cells were resuspended. A small drop of fine bone marrow cell smears was prepared from the final cell suspensions on clear glass slides. After air-drying (2–4 h) at room temperature, slides were stained using routine May-Grünwald–Giemsa staining method.

Analysis of differential blood count and myelogram was done on the microscope (Eclipse E600, Nikon, Amsterdam, The Netherlands) by using an immersion lens (1000×) and by counting at least 500 cells.

The bone marrow was analyzed by determination of the quantitative relationship of granulopoiesis in comparison with erythropoiesis, as well as by the percentage representation of individual subpopulations of white blood cells. Also, a relative number of immature (myeloblasts and promyelocytes) and intermediate (myelocytes, metamyelocytes and non-segmented granulocytes) leukocytes, between all experimental groups, was compared. The second group for comparison consisted of mature leukocytes (neutrophils and eosinophils). As a group called “eosinophils”, we considered cells with eosinophilic granules, immature as well as mature forms. Therefore, precursors of eosinophils have not been included in the subpopulation of myelocytes and metamyelocytes.

### 4.7. Histopathological Study and Semiquantitative Analysis

In order to evaluate the hepatoprotective and nephroprotective effects of AMI, ten animals from each experimental group were sacrificed 28 days after receiving their last treatment under light ether anesthesia. At necropsy, the dissected liver and kidney tissue was carefully spread over a metal tray coated with wax and fixed with 10% neutral buffered formalin solution. Five to seven days after fixation, all tissues were divided into four portions in order to be prepared for making sections. After the fixation process, all tissue samples were dehydrated in graded alcohol (100%, 96%, and 70%), xylol, and embedded in paraffin blocks. Finally, 2 μm-thick paraffin sections were stained by hematoxylin and eosin (H&E) method, and whole visual fields magnified by 200× were analyzed (Olympus BX 43, Olympus, Tokyo, Japan).

The type, degree, and severity of hepatic and renal lesions, along with the degree of inflammatory cellular infiltration, were assessed in all tissue sections from each animal, and they were counted in separate visual fields at 400× magnification (Olympus BX 43, Olympus, Japan). The severity of hepatic and renal lesions consisting of edema, cellular infiltration, hemorrhages, vacuolar degeneration, necrosis, and the distribution of lesions (e.g., focal, multifocal, locally extensive, or diffuse) were assessed and graded by two independent pathologists. From each slice, whole visual fields were analyzed by using light microscope according to the 5-point semiquantitative scale previously described [32,73]. A severity grade was expressed as hepatic damage score (HDS) and renal damage score (RDS), and the exact method of calculation is shown in Table 4 and Table 5, respectively.

### 4.8. Statistical Analysis

Complete statistical analysis of data was done with the statistical software package (Stat for Windows, R.7, Stat Soft, Inc., San Francisco, CA, USA, 2008). In the case of continuous data, variables were presented as mean value ± standard deviation (SD). Kolmogorov–Smirnov test was used for evaluation of normality of presented data. If data were normally distributed, 1-way ANOVA and post hoc Bonferroni tests were used. If data were not normally distributed, Kruskal–Wallis 1-way ANOVA and post hoc Mann–Whitney *U* tests were used. All the analyses were estimated at minimal *p* < 0.05 level of statistical significance.

## 5. Conclusions

Based on these findings, it can be concluded that AMI administered at a dose of 75 mg/kg 30 min before DOX significantly attenuated the effects of its cumulative dose (20 mg/kg body weight for 28 days) in rats, concerning animals’ mortality, body, liver and kidney weight, as well as structural alterations of the bone marrow, liver and kidney. In addition, this study showed that AMI, given in a dose of 75 mg/kg body weight, four times per week, for four weeks, is not toxic, *per se*.

Successful protection of rats by AMI against multiple-dose DOX-induced toxicity is mediated by complex, and still not fully elucidated mechanisms of action.

## Figures and Tables

**Table 1:** The influence of amifostine and/or doxorubicin on relative (%) liver and kidney weight of the rats 4 weeks after receiving the last treatment.

Treatment	Liver (%)	Kidney (%)
Control	2.79 ± 0.36	0.26 ± 0.02
AMI	2.86 ± 0.30	0.26 ± 0.01
DOX	3.36 ± 0.22 **a^1^**	0.37 ± 0.08 **a^1^**
AMI + DOX	3.29 ± 0.36	0.31 ± 0.01

Statistical analysis was performed using Bonferroni test. The results are expressed as the percentage from the control group on day 0. **a^1^**—*p* < 0.05 in comparison to the control group.

**Table 2:** The influence of different treatment on myelogram (%) in the bone marrow 28 days after receiving last treatment.

Parameter	Control	AMI	DOX	AMI + DOX
Myeloblasts	0.55 ± 0.41	0.54 ± 0.60	0.70 ± 0.20	0.53 ± 0.82
Promyelocytes	3.05 ± 0.41	3.21 ± 1.14	2.90 ± 1.23	3.56 ± 0.97
Myelocytes	5.27 ± 0.47	5.61 ± 1.55	6.22 ± 1.61	5.58 ± 1.82
Metamyelocytes and band cells	14.64 ± 6.35	12.65 ± 5.42	6.90 ± 3.13 **a^1^**	10.68 ± 2.05 **b^1^**
Neutrophils	41.32 ± 3.81	44.07 ± 6.59	37.15 ± 4.19	45.63 ± 3.42 **b^2^**
Eosinophils	8.35 ± 4.59	10.60 ± 3.91	19.45 ± 4.86 **a^1^**	12.36 ± 3.68 **b^1^**
Lymphocytes	23.05 ± 9.74	19.77 ± 8.68	20.76 ± 8.98	17.95 ± 4.32
Monocytes	3.32 ± 1.42	2.31 ± 0.88	4.05 ± 1.47	2.65 ± 1.65
Plasma cells	1.07 ± 1.35	1.58 ± 0.44	1.55 ± 1.51	0.91 ± 0.84
Mast cells	0.50 ± 0.52	0.00 ± 0.00	0.07 ± 0.15 **a^1^**	0.00 ± 0.00 **a^1^**
White/red cell line	2.15 ± 0. 56	2.65 ± 0.42	2.04 ± 0.86	2.65 ± 0.93 **a^2^**

Statistical analysis was performed using Bonferroni test. The results are expressed as a percentage related to the control group on day 0. **a^1^**, **a^2^**—*p* < 0.05, 0.01 in comparison to the control group; **b^1^**, **b^2^**—*p* < 0.05, 0.01 in comparison to the DOX-only treated group.

**Table 3:** The influence of different treatment on differential blood count (%) in the peripheral blood 28 days after receiving last treatment.

Parameter	Control	AMI	DOX	AMI + DOX
Neutrophils	19.40 ± 5.02	20.00 ± 4.60	14.00 ± 6.16	24.33 ± 4.76 **b^1^**
Eosinophils	1.80 ± 0.83	3.66 ± 4.58	3.00 ± 1.82	1.66 ± 1.21
Lymphocytes	71.40 ± 8.70	70.00 ± 3.03	67.25 ± 10.68	60.00 ± 6.41 **a^1^**
Monocytes	7.40 ± 4.03	8.00 ± 3.03	15.75 ± 4.19 **a^1^**	14.00 ± 4.14 **a^1^**

Statistical analysis was performed using Bonferroni test. The results are expressed as a percentage related to the control group on day 0. **a^1^**—*p* < 0.05 in comparation to the control group; **b^1^**—*p* < 0.05 in comparation to the DOX-only treated group.

**Table 4:** The effects of different treatments on the degree of hepatic alterations 28 days after receiving last treatment.

Treatment (mg/kg)	Hepatic Damage Score (HDS) (10 Livers/Group × 4 Slices/Liver)						$\bar{\times }\;\pm \;S.D.$
	0	1	2	3	4	5	
1. Control	30	10	0	0	0	0	0.25 ± 0.46
2. AMI	0	25	15	0	0	0	1.37 ± 0.75 **a^2^**
3. DOX	0	0	0	0	15	25	4.62 ± 0.51 **a^3^** **b^3^**
4. AMI + DOX	0	0	0	15	25	0	3.62 ± 0.51 **a^3^** **b^3^** **c^1^**

Statistical analysis was performed using a Kruskal–Wallis test. **a^2^**, **a^3^**—*p* < 0.01, 0.001 in comparison to the control group, **b^3^**—*p* < 0.001 in comparison to the AMI-only treated group, **c^1^**—*p* < 0.05 in comparison to the DOX-only treated group.

**Table 5:** The effects of different treatments on the degree of renal alterations 28 days after receiving last treatment.

Treatment (mg/kg)	Renal Damages Score (RDS) (10 Kidneys/Group × 4 Slices/Kidney)						$\bar{\times }\;\pm \;S.D.$
	0	1	2	3	4	5	
1. Control	30	10	0	0	0	0	0.25 ± 0.46
2. AMI	0	20	20	0	0	0	1.50 ± 0.53 **a^3^**
3. DOX	0	0	0	0	10	30	4.75 ± 0.46 **a^3^** **b^3^**
4. AMI + DOX	0	0	0	30	10	0	3.25 ± 0.46 **a^3^** **b^3^** **c^2^**

Statistical analysis was performed using a Kruskal–Wallis test. **a^2^**, **a^3^**—*p* < 0.01, 0.001 in comparison to the control group, **b^3^**—*p* < 0.001 in comparison to the AMI-only treated group, **c^2^**—*p* < 0.01 in comparison to the DOX-only treated group.
